# Economic Evaluation of the Next Generation Electronic Medical Records in Singapore: Cost-Utility Analysis

**DOI:** 10.2196/70484

**Published:** 2025-06-11

**Authors:** Cynthia Chen, Jarawee Sukmanee, Khai Wee Soon, Julian Lim, Jared Louis Andre D'Souza, Yot Teerawattananon

**Affiliations:** 1 Saw Swee Hock School of Public Health National University Health System National University of Singapore Singapore Singapore; 2 Schaeffer Center for Health Policy and Economics University of Southern California Los Angeles United States; 3 Health Intervention and Technology Assessment Program Ministry of Public Health Nonthaburi Thailand; 4 National University Polyclinics Singapore Singapore Singapore; 5 Ng Teng Fong General Hospital Singapore Singapore

**Keywords:** electronic medical records, cost-effectiveness, cost-utility, ICER, primary care, QALY, incremental cost-effectiveness ratio, quality-adjusted life year

## Abstract

**Background:**

With the vast development of technology and the evolving needs of patients and health care providers, electronic medical records (EMRs) have become a cornerstone for health information. However, different institutions have used different EMR systems. Our study investigates the potential benefits of implementing an integrated and common platform, known as the Next Generation Electronic Medical Record (NGEMR) in Singapore. The NGEMR allows improved data sharing between health care facilities and can promote better coordination between primary care and specialist care doctors to access patients’ records from the same database.

**Objective:**

This study aims to conduct an economic evaluation of the NGEMR to inform future health care system upgrades.

**Methods:**

A cost-utility analysis comparing NGEMR with the legacy EMR was conducted using a decision tree model with a 1-year time horizon from a health care system perspective. Input parameters of patients visiting primary care at the National University Polyclinics and specialist outpatient clinics from a General Hospital were extracted from the EMR systems. The incremental cost-effectiveness ratio (ICER) was calculated using costs and quality-adjusted life years (QALYs).

**Results:**

NGEMR was cost-effective and yielded a marginal health benefit (0.00006 QALYs gained) at a slightly higher cost (S $2.73; US $2.02), with an ICER of S $46,349 (US $34,298) per QALY. At the willingness-to-pay thresholds of 0.5- and 1-time gross domestic product (GDP) per capita (S $48,899; US $36,185 and S $97,798; US $72,371 per QALY), the implementation of NGEMR had a 52.2% and 64.7% probability of being cost-effective, respectively. The reduction in waiting time to see a specialist resulted in 2.3% fewer hospitalizations. The most influential parameter on the ICER was the probability of receiving duplicate tests, followed by the costs of admission and the probability of seeing a specialist. Reducing the probability of receiving duplicate tests for NGEMR from 20.7% to 13.2% resulted in a cost-saving ICER. A threshold analysis on the proportion of patients with a waiting time of less than 20 days for NGEMR was further explored, as it was a sensitive parameter on the cost-effectiveness of NGEMR. Increasing the proportion of patients with a waiting time of less than 20 days from 45.5% to 56% would result in cost savings for NGEMR.

**Conclusions:**

The adoption of NGEMR is cost-effective in Singapore. Beyond cost-effectiveness, the reduction of waiting time between primary and specialist care can lower the possibility of patients’ health deterioration, thus reducing hospital admissions. We recommend continuous monitoring of waiting times and the likelihood of having duplicate tests as countries transition from basic to advanced-level EMR systems. Future analyses could benefit from more granular data on timing and clinical indications and incorporate real-world local data as they become available through ongoing NGEMR rollout evaluations.

## Introduction

With the vast development of technology and the complex needs of patients and health care providers, electronic medical records (EMRs) have provided a platform for health information storage and are accessed only by authorized people [1]. The lack of health information can cause delays and increased health care expenses [2]. Thus, the implementation of EMRs has a huge potential to improve patients’ outcomes.

Singapore’s health care delivery system can be categorized into primary, acute, and long-term care. Primary care includes public polyclinics and private general practitioner clinics. Acute care consists of hospitals and specialist outpatient clinics (SOCs) in the private and public sectors. Finally, long-term care consists of nursing homes and community hospitals [3]. The median waiting time to see a specialist ranged between 24 to 35 days from January to November 2020 [4].

In 2011, Singapore started the National Electronic Health Records system to share medical records across health care establishments. The system uses information contributed by the participating institutions’ EMRs to facilitate exchanging crucial patient information, including diagnoses, treatments, tests, and medical histories.

However, health care providers have limited access to the case notes from the institutions’ EMRs. They face difficulties accessing more detailed information from different health care providers due to the lack of an integrated platform [5]. This can lead to, for example, duplication of tests from different health care facilities and unnecessary health care expenses, when patients are referred from primary to secondary or tertiary care. Care coordination failure can be costly and has been estimated to cost between US $27.2 billion and US $78.2 billion in the United States [6]. As such, when patients are managed seamlessly across health care facilities, it helps to reduce costs to the health care system [7].

Hence, intending to integrate medical records from various health care institutions, the Singapore Ministry of Health has implemented a nationwide Next Generation Electronic Medical Record (NGEMR) system in different phases [8] from 2018 onwards, aiming to facilitate the provision of high-quality care management and delivery, foster stronger collaborations among health care professionals, and enable the collection of more comprehensive information for medical education and research efforts. As of July 2024, the NGEMR system has been implemented in 37 health care institutions across 2 of the 3 regional health clusters, namely the National University Health System and National Healthcare Group, whereas the implementation of the NGEMR system in the last cluster, Singhealth, will take place from 2026 to 2028 [9,10]. The NGEMR documents the entire patient journey, including admission, discharge, and outpatient clinic visits, recording medical and administrative data. Therefore, with the NGEMR system, improved data sharing between health care facilities can promote more effective and personalized patient care, as well as better coordination between primary, secondary, and tertiary care. This can also help to reduce the waiting time from primary to specialist care, consequently reducing patients’ missed appointments with specialists [11,12]. Likewise, the continuity of primary and specialty care is associated with a decreased risk of an emergency department (ED) visit and hospital admission [13], potentially resulting in reduced health care costs.

While EMRs have been widely adopted globally, the transition to more advanced systems such as Singapore’s NGEMR aims to enhance interoperability, data integration, and care continuity. However, there is limited evidence of the economic impact of this transition, particularly in the Singapore context. To our knowledge, no cost-effectiveness analysis has been conducted yet to compare the current EMR system with the NGEMR, leaving a critical gap in understanding the value and return on investment for health care institutions and policy makers. The majority of existing literature on economic evaluation primarily compares basic EMRs with traditional paper-based systems [14]. These cost-benefit studies conclude that basic EMRs can modestly improve the quality of life without substantially increasing health care costs [15] and yield a positive net financial return on investment [16-18]. A recent systematic review reveals that advanced-level EMRs do not significantly lower costs [19]. Nevertheless, advanced-level EMRs are defined as systems with computerized provider order entry and clinical decision support, which differs from the integrated platform provided by NGEMR. While earlier studies, such as the systematic review [18], concluded that EMRs may not significantly reduce costs in primary care settings, these findings may not fully apply to newer, integrated systems such as NGEMR. Unlike earlier EMRs, which were often limited to individual clinics or institutions, NGEMR is designed to support system-wide interoperability and coordinated care across multiple levels of the health care system. Thus, it remains unclear if implementing NGEMR in Singapore will significantly lower health care costs, improve quality of life, and yield a greater financial return on investment. Therefore, this study aims to assess the cost-effectiveness of NGEMR, which will be useful for informing future investments in health care system upgrades.

## Methods

### Overview

A model-based economic evaluation was conducted from the health care system perspective to compare the previous EMR system to NGEMR. Multimedia Appendix 1 summarizes the core differences between EMR and NGEMR, with the latter system allowing doctors from both primary and specialist care to access shared patients’ records. Expected outcomes were computed in quality-adjusted life years (QALYs) to reflect life expectancy and quality of life effects. We evaluated the costs and outcomes over a 1-year time horizon, therefore, no discounting is required. This study followed the Consolidated Health Economic Evaluation Reporting Standards 2022 (CHEERS 2022) [20], as shown in Multimedia Appendix 2.

### Model Structure

A decision tree model was developed using TreeAge Pro 2023 software (TreeAge Software Inc). The model was developed using a literature review and in close consultation with clinical stakeholders, including physicians, heads of the department, and health informatics specialists, to ensure it accurately represents real-world workflows associated with EMR and NGEMR use in hospital and primary care settings. Key clinical pathways, documentation processes, and decision points were mapped to reflect patients’ encounters. The model underwent face validation through iterative discussions with clinicians and informaticians, and internal validation was conducted to ensure logical consistency and appropriate flow of probabilities. Calibration was performed by comparing model outputs with observed hospital and operational data, where available. This analysis adopts a variable cost perspective, focusing on recurring costs through bill sizes of EMR versus NGEMR postimplementation. Capital expenditures related to system procurement, infrastructure setup, and migration were excluded. This is consistent with Singapore’s current health IT strategy, which involves a full-scale national rollout of NGEMR across public health care institutions [10]. Thus, comparing variable costs through bill sizes is more relevant for supporting institutional resource planning and postimplementation evaluation.

After analyzing EMR and NGEMR databases, we observed a decline in waiting time to see a specialist after the implementation of NGEMR (Multimedia Appendix 3). Therefore, our model was constructed based on the assumption that NGEMR would enhance accessibility to specialist care, as measured by a waiting time of less than 20 days. Additionally, patients were more likely to follow up with their specialists if their waiting time was less than 20 days (EMR 86.6% vs NGEMR 88%); thus, the probability of seeing a specialist varied by waiting times (ie, <20 days or ≥20 days). After seeing a specialist, there was a probability that patients could receive duplicate tests [21]. We assumed that the probability of receiving duplicate tests would be reduced with NGEMR (EMR 29.4% vs NGEMR 20.7%) [21]. Regardless of the duplicate test status, patients could be admitted after seeing a specialist. We also assumed that timely management of chronic diseases at the specialist clinic could reduce severe complications, resulting in a slight reduction in the probability of hospital admission (EMR 11.83% vs NGEMR 11.55%) [22,23]. These improvements would increase QALYs, as admitted patients had lower QALYs compared with nonadmitted patients (EMR 0.89 vs NGEMR 0.91) [24]. Patients who did not see a specialist could visit the ED and be admitted to the hospital, or they could remain healthy without requiring an ED visit.

### Input Parameters

Input parameters of patients visiting primary care at the 5 polyclinics under the National University Polyclinics (NUP) and the SOCs at Ng Teng Fong General Hospital (NTFGH) were extracted from the EMR systems. These polyclinics represent one of the 3 regional health clusters (National University Health System) in Singapore. Since the NUP and hospital-based SOCs were not directly linked at the patient level, we relied on referral protocols and patterns validated through expert consultations with physicians and care coordinators to model patient transitions between primary and specialist care. While exact patient-level transitions could not be traced, probabilities of referral and follow-up were informed by aggregate data from local settings [22-25], where possible. Characteristics of patients visiting primary care at NUP and specialist care at NTFGH, stratified by period, are provided in Multimedia Appendix 4. Missing parameters were referenced from national and international literature, as shown in Table 1.

**Table 1:** Parameters used in the decision tree model.

Parameter			Mean		SE		Distribution		Reference
**Probability**									
	Proportion of patients with a waiting time of <20 days, EMR^a^	0.37		0.004		Beta		SOCs^b^ dataset	
	Proportion of patients with a waiting time of <20 days, NGEMR^c^	0.46		0.004		Beta		SOCs dataset	
	Probability of seeing a specialist given a referral appointment in patients with a waiting time of <20 days, EMR	0.87		0.005		Beta		SOCs dataset	
	Probability of seeing a specialist given a referral appointment in patients with a waiting time of ≥20 days, EMR	0.82		0.004		Beta		SOCs dataset	
	Probability of seeing a specialist given a referral appointment in patients with a waiting time of <20 days, NGEMR	0.88		0.004		Beta		SOCs dataset	
	Probability of seeing a specialist given a referral appointment in patients with a waiting time of ≥20 days, NGEMR	0.80		0.004		Beta		SOCs dataset	
	Probability of receiving duplicate tests after seeing a specialist, EMR	0.29		0.05		Beta		[21]	
	Probability of receiving duplicate tests after seeing a specialist, NGEMR	0.21		0.04		Beta		[21]	
	Probability of admission after seeing a specialist, EMR	0.12		0.002		Beta		[22]	
	Probability of admission after seeing a specialist, NGEMR	0.12		0.002		Beta		[23]	
	Probability of ED^d^ visit and admission	0.30		0.002		Beta		[25]	
**Direct medical costs (**S$**)**									
	Costs of NUP^e^ visit, EMR	102.30 (US $75.70)		0.25		Gamma		NUP dataset	
	Costs of NUP visit, NGEMR	114.73 (US $84.90)		0.28		Gamma		NUP dataset	
	Costs of SOCs visit without duplicate tests, EMR	189.07 (US $139.91)		0.92		Gamma		SOCs dataset	
	Costs of SOCs visit without duplicate tests, NGEMR	191.67 (US $141.84)		1.09		Gamma		SOCs dataset	
	Costs of SOCs visit with duplicate tests, EMR	266.04 (US $196.87)		0.94		Gamma		Assumption	
	Costs of SOCs visit with duplicate tests, NGEMR	270.41 (US $200.10)		1.12		Gamma		Assumption	
	Costs of admission	2707 (US $2,002.18)		Shape=2.06 and Scale=1549.32		Gamma		[26]	
	Costs of ED visit	141 (US $104.34)		—		—		[27]	
**Utility**									
	QALYs^f^ in admitted patients	0.89		0.019		Beta		[28]	
	QALYs in nonadmitted patients	0.91		0.01		Beta		[24]	

^a^EMR: electronic medical records.

^b^SOC: specialist outpatient clinic.

^c^NGEMR: Next Generation Electronic Medical Records.

^d^ED: emergency department.

^e^NUP: National University Polyclinics.

^f^QALY: quality-adjusted life year.

**Figure 1:**
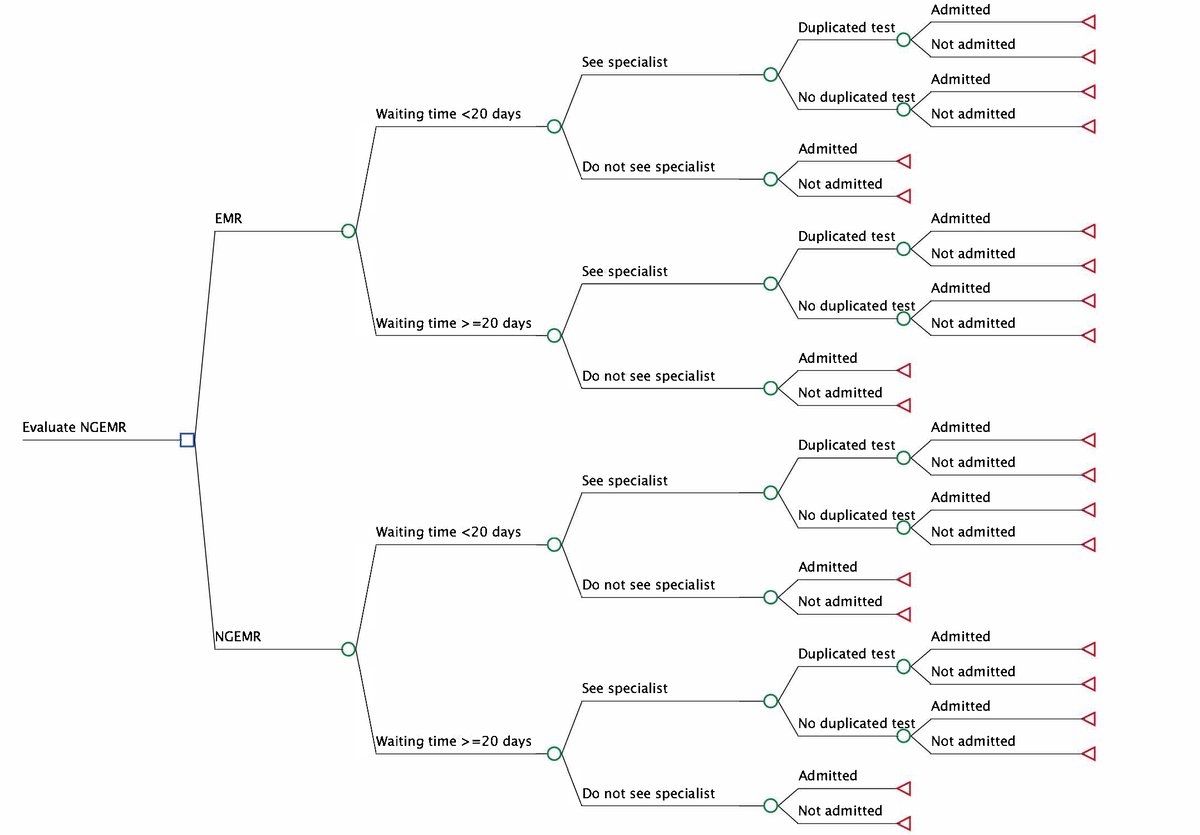
Decision Tree Model. The figure shows the decision tree model used to represent real-world workflows associated with EMR and NGEMR use in hospital and primary care settings. EMR: electronic medical record; NGEMR: Next Generation Electronic Medical Record.

Distributions were selected based on the nature and characteristics of each parameter [29], following standard conventions in health economic modeling. Parameters bounded between 0 and 1 (eg, probabilities and utilities) were assigned beta distributions, which are well-suited for modeling values within this range. Cost parameters, which are continuous and positively skewed, were modeled using gamma distributions. The mean and SE for each parameter were obtained either from our data analysis or from published literature and were used to parameterize the respective distributions. The proportion of patients with a waiting time of less than 20 days and the probability of seeing a specialist by waiting time were derived from the primary and specialist care datasets. Several assumptions were necessary to operationalize the decision tree model (Figure 1), including those related to waiting times, patterns of resource utilization and associated costs, and the likelihood of receiving duplicate tests, among others. These assumptions were informed by existing literature and further validated through expert consensus. For instance, duplicate testing is defined by Rome et al [21] as the repetition of a diagnostic test within 48 hours of an initial test when a patient transitions between health care facilities. This duplication occurs on the same body part and uses similar testing methodologies, typically due to the unavailability of previous test records. In the absence of empirical data on duplicate test rates from Singapore’s public health care system, we relied on published estimates from international studies comparing nonintegrated and integrated EMR systems. We conservatively assumed that the probability of receiving duplicate tests was 30% lower during NGEMR, based on estimates from another study [21]. In addition, due to the lack of inpatient data, the probability of hospital admission was obtained from the general population in Singapore from 2020 to 2021 [22,23]. The probability of ED visits and admissions was based on a previous study in Singapore [25]. As mentioned in the *Methods* section above, we assumed that timely management of chronic diseases at the specialist clinic could reduce severe complications, resulting in a slight reduction in the probability of hospital admission (EMR 11.83% vs NGEMR 11.55%) [22,23].

The costs of primary care and specialist visits without duplicate tests were calculated using the NUP and SOCs datasets, respectively. Due to unbalanced patient characteristics during the pre- and post-NGEMR periods (Multimedia Appendix 2), patients visiting NUP in different periods were matched by type of visit (ie, acute or chronic) before calculating costs. The health care costs of SOC visits in patients with duplicate tests were assumed to be S$50 (US $37) higher than those without duplicate tests, based on the unsubsidized health screening costs in Singapore (eg, full blood count and electrocardiogram) [30,31]. As hypertension was the most common diagnosis in the NUP dataset, the costs of admission for patients with high blood pressure without severe complications were retrieved from the Ministry of Health’s hospitalization database [26]. The costs of ED visits were based on NTFGH’s services and facility fees for Accident and Emergency [27]. All costs were reported in 2021 Singapore dollars (S$), where US $1 is equivalent to S$1.35 in December 2021 [32].

We used the mean EQ-5D score, measured using Singapore preference weights in patients with hypertension from a previous study [24], as the QALYs of nonadmitted patients. Since there was no data on QALYs for Singapore patients admitted with hypertension, we derived the QALYs for admitted patients by multiplying the QALYs of nonadmitted patients by the percentage drop in utility observed in patients admitted with chronic heart failure in a European study [28].

### Cost-Effectiveness Analysis and Uncertainty Analysis

The cost-effectiveness was assessed using the incremental cost-effectiveness ratio (ICER). Based on a recent systematic review [33], the willingness-to-pay threshold was set at S $48,899 (US $36,185.26) per QALY, which was 0.5 times the gross domestic product (GDP) per capita in 2021 for Singapore [34]. We also explored the cost-effectiveness at the willingness-to-pay threshold of S $97,798 (US $72,370.52) per QALY (1 times the GDP per capita). The probabilistic sensitivity analysis (PSA) was performed using 1000 Monte Carlo simulations. To assess the robustness of the assumptions and their influence on model outcomes, plausible parameter estimates were subjected to one-way and scenario-based sensitivity analyses, thereby evaluating the impact of uncertainty in key parameters on the model’s predictions. The PSA results were presented as scatter plots and cost-effectiveness acceptability curves. For the 1-way sensitivity analysis, the upper and lower bounds were set at 95% CI, except for the costs of admission, where the SD was unavailable, and the bounds were based on the IQR. The results were displayed in a tornado diagram.

### Ethical Considerations

This study was approved by the National Healthcare Group (NHG) Domain Specific Review Board (DSRB) (reference number: 2020/00156). The IRB approval covers secondary analysis without additional content. This research used deidentified datasets and does not involve human subjects or human biological materials.

## Results

### Base Case

Table 2 shows the results of the base-case analysis. The one-year implementation of NGEMR provided a small health benefit (0.00006 QALYs gained) at a slightly higher cost (S $2.73; US $2.02) compared with the pre-NGEMR period. As a result, the ICER for NGEMR was calculated to be S $46,349 (US $34,298) per QALY, which is considered cost-effective at the willingness-to-pay threshold of S $48,899 (US $36,185) per QALY.

**Table 2:** Costs, outcomes, and incremental cost-effectiveness ratio (ICER) for Next Generation Electronic Medical Records (NGEMR).

Indicator	Comparator (pre-NGEMR^a^)	Intervention (post-NGEMR)
Costs (S$)	687.80 (US $508.97)	690.52 (US $511.99)
QALYs^b^	0.90647	0.90653
Incremental costs (S$)	—^c^	2.73 (US $2.02)
Incremental QALYs	—	0.00006
ICER^d^ (S$ per QALY)	—	46,349 (US $34,298)

^a^NGEMR: Next Generation Electronic Medical Records.

^b^QALY: quality-adjusted life year.

^c^Not applicable.

^d^ICER: incremental cost-effectiveness ratio.

### Uncertainty Analysis

**Figure 2:**
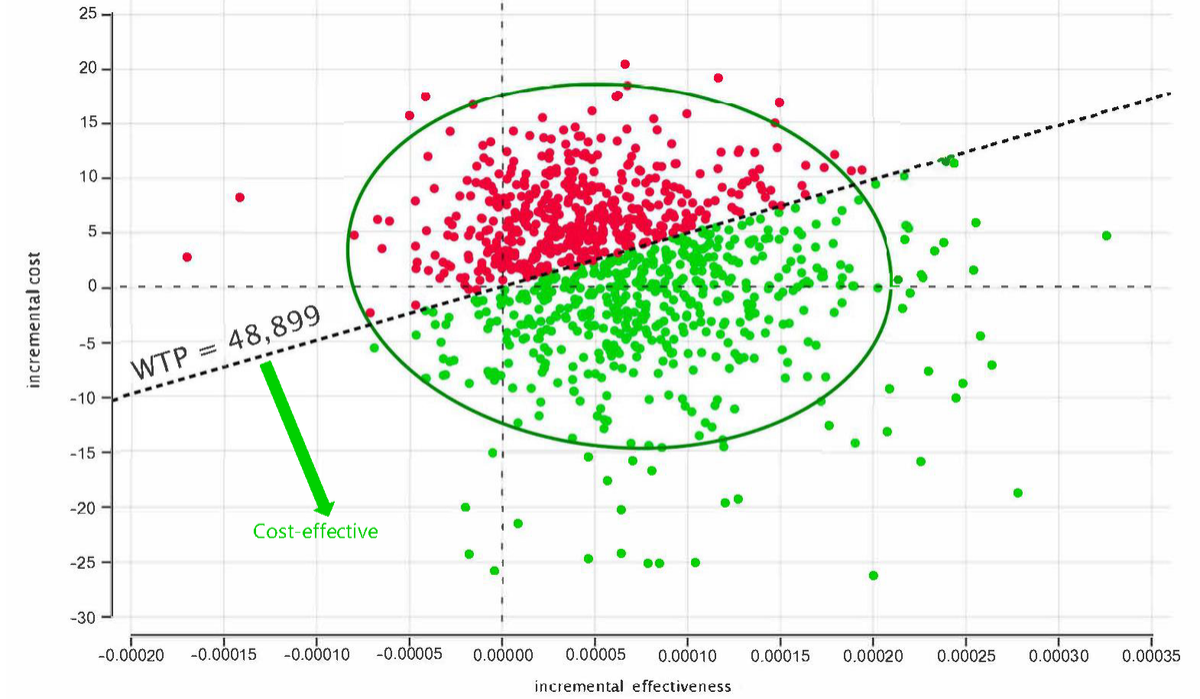
Cost-effectiveness plane showing results from 1000 simulations of probabilistic sensitivity analysis. Each dot represents a single run of the Monte Carlo simulation. Green dots indicate the cost-effectiveness of Next Generation Electronic Medical Record implementation at willingness-to-pay thresholds of S $48,899 (US $36,222.96) per quality-adjusted life year. EMR: electronic medical record; NGEMR: Next Generation Electronic Medical Record; PSA: probabilistic sensitivity analysis; WTP: willingness to pay.

**Figure 3:**
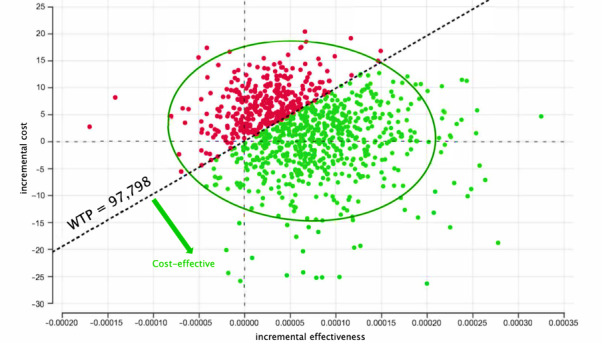
Cost-effectiveness plane showing results from 1000 simulations of probabilistic sensitivity analysis. Each dot represents a single run of the Monte Carlo simulation. Green dots indicate the cost-effectiveness of Next Generation Electronic Medical Record implementation at willingness-to-pay thresholds of S$97,798 (US $72,445.89) per quality-adjusted life year. EMR: electronic medical record; NGEMR: Next Generation Electronic Medical Record; PSA: probabilistic sensitivity analysis; WTP: willingness to pay.

**Figure 4:**
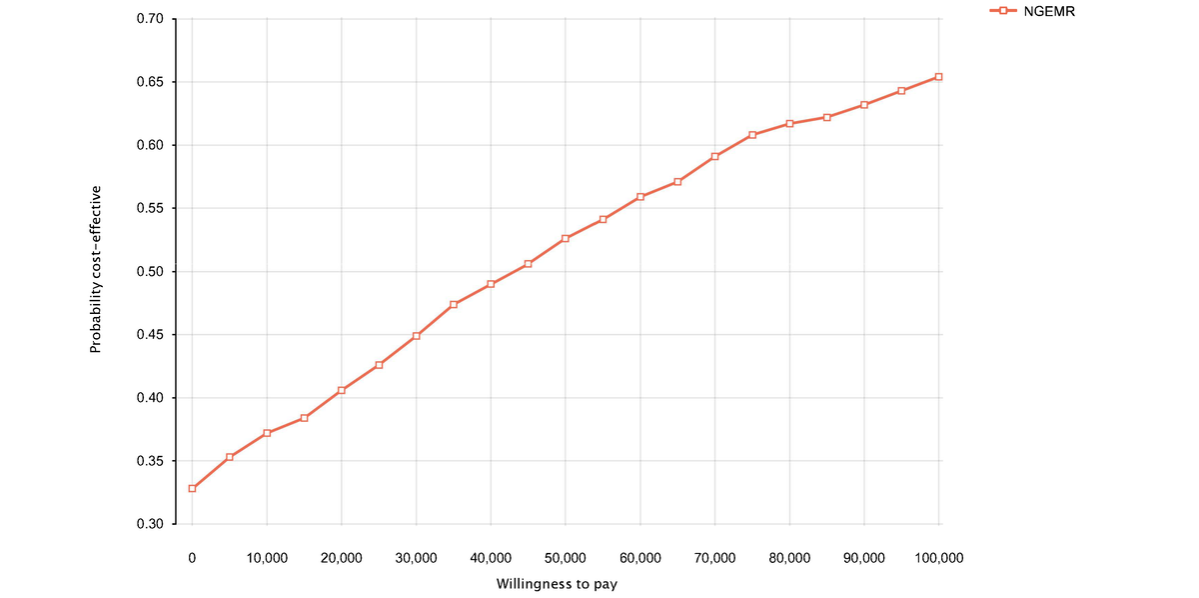
Cost-effectiveness acceptability curve showing the probability of Next Generation Electronic Medical Record being cost-effective at different willingness-to-pay thresholds. NGEMR: Next Generation Electronic Medical Record.

Results from the PSA were presented as cost-effectiveness planes and cost-effectiveness acceptability curves in Figures 2, 3, and 4, respectively. The scatterplot of 1000 simulations on the cost-effectiveness plane was mostly clustered in the upper-right quadrant (Figures 2 and 3), indicating that the implementation of NGEMR was more expensive and more effective. At the willingness-to-pay thresholds of 0.5 times (S $48,899; US $36,223) and 1 times GDP per capita (S $97,798; US $72,446) per QALY, the implementation of NGEMR had a 52.2% and 64.7% probability of being cost-effective, respectively (Figure 4).

**Figure 5:**
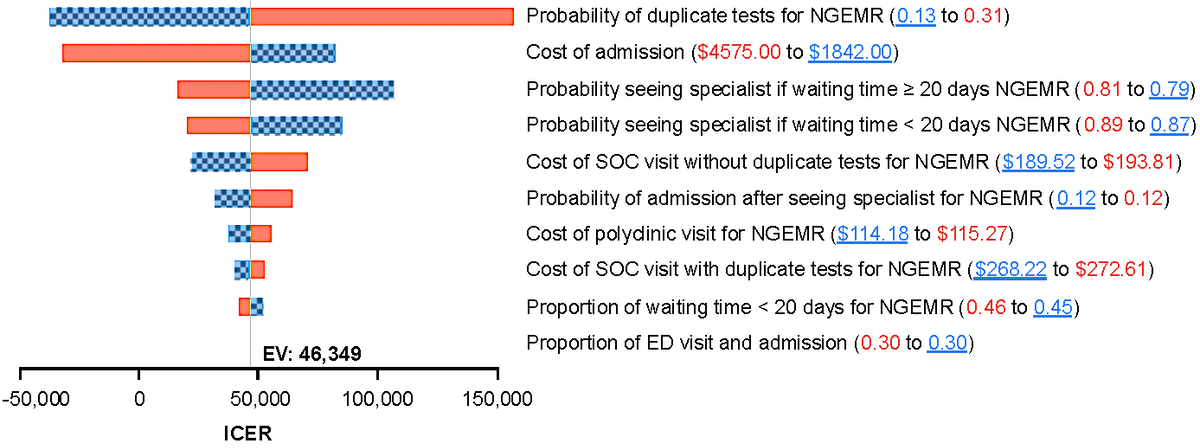
Tornado diagram showing the parameters impacting the incremental cost-effectiveness ratio in descending order. The checkered bar shows the incremental cost-effectiveness ratio range due to the change in each parameter, as shown by the underlined value. ED: emergency department; EMR: electronic medical record; EV: expected value; ICER: incremental cost-effectiveness ratio; NGEMR: Next Generation Electronic Medical Record; SOC: specialist outpatient clinic.

**Figure 6:**
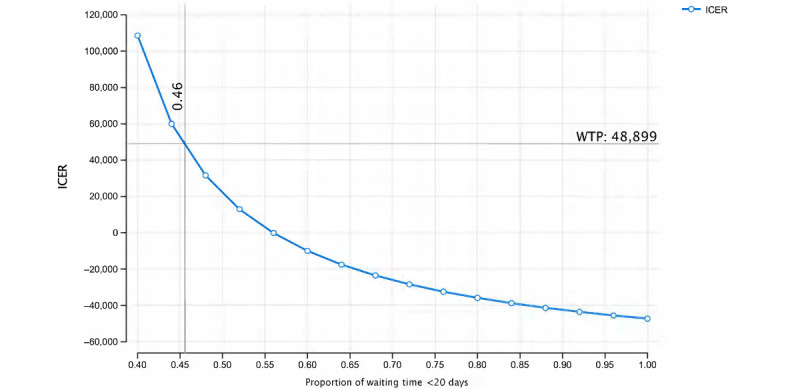
Threshold analysis on the proportion of patients with a waiting time of less than 20 days. EMR: electronic medical record; ICER: incremental cost-effectiveness ratio; NGEMR: Next Generation Electronic Medical Record; WTP: willingness to pay.

The tornado diagram for one-way sensitivity analysis is illustrated in Figure 5. The most influential parameter was the probability of receiving duplicate tests, followed by the costs of admission and the probability of seeing a specialist. Reducing the probability of receiving duplicate tests for NGEMR from 20.7% (base-case analysis) to 13.2% (lower bound of 95% CI) resulted in a cost-saving ICER. A threshold analysis on the proportion of patients with a waiting time of less than 20 days for NGEMR was further explored, as it was a sensitive parameter in the cost-effectiveness of NGEMR. Increasing the proportion of patients with a waiting time of less than 20 days from 45.5% to 56% would result in cost savings for NGEMR (Figure 6).

## Discussion

### Principal Findings

Our findings indicate that the implementation of NGEMR provides a small health benefit at a slightly higher cost. The return on investment for NGEMR highly depends on reducing the proportion of duplicate tests. For instance, if the probability of duplicate tests for NGEMR is reduced from 20.7% to less than 13.2%, NGEMR can yield cost savings. Furthermore, the reduction in waiting time between primary and specialist care increases the probability of patients seeing a specialist. Our sensitivity analysis shows that if the percentage of patients who experience a waiting time of less than 20 days is increased from 45.5% to 56%, NGEMR can result in cost savings. A less than 20-day waiting time is not an impossible target, as the median waiting time to see a specialist ranged between 24 to 35 days from January to November 2020 [4]. During this period, backlogs from the COVID-19 circuit breaker period were cleared as quickly as possible [4]. Results from our sensitivity analysis suggest that if better patient management at the SOC can lead to fewer hospitalizations, NGEMR will be cost-saving.

With a willingness-to-pay threshold of S $48,899 (0.5 of GDP; US $36,222.96), we find that NGEMR is cost-effective with an ICER of S $46,349 (US $34,333) per QALY. This is similar to the findings from a UK study, which demonstrated the cost-effectiveness of an EMR-based clinical decision support system for adults with diabetes [15]. The study shows an ICER of US $65,459 per QALY when the intervention effect persists for one year. Given that the NGEMR is implemented to enhance the quality of patient care [8], our model assumed the benefit of the NGEMR in reducing wait times and duplicate tests. With a reduction in waiting time, patients have higher follow-up rates with their specialists. This could lower the possibility of patient health deteriorating too rapidly, as patients are better managed in an outpatient setting [35] and are less likely to be admitted to the ED and hospital. A recent systematic review also concluded that of the 15 studies, 93.3% demonstrated that higher continuity of care in the outpatient setting was significantly associated with fewer hospitalizations for ambulatory care-sensitive conditions [36]. This can further reduce additional pressure on hospital beds and costs on hospitals [37].

To the best of our knowledge, there is no existing literature that evaluates the effect of reducing waiting time to see a specialist on cost and patient outcomes. Our study provides some insights into how the NGEMR improves health outcomes per unit cost compared with basic EMR. This is crucial to answering the research question of whether an EMR upgrade is cost-effective. In our decision analytic model, we used conservative estimates for our annual transitional probabilities, QALYs, and costs. QALY values were also obtained from EQ-5D data that were specifically based on the Singaporean population [24]. Our probability of admission was based on the latest statistical data reflected on the Singapore Ministry of Health’s official website [22,23]. We also used conservative cost estimates by using median costs from the official Ministry of Health fee benchmarks and public health care institutions [26]. These direct medical costs are fundamental to the patient experience and do not include indirect benefits of wait time reduction, such as the median wages due to the reduced number of hospitalizations.

### Limitations

There are several limitations in our dataset. The analysis adopts a 1-year time horizon, aligning with annual budget cycles and short-term operational planning commonly used in health care system evaluations. However, this timeframe may underestimate the full impact of NGEMR, particularly its potential to improve chronic disease management, reduce hospital readmissions, and generate long-term cost savings through better care coordination. Future research should consider extended time horizons and dynamic modeling approaches, using Markov simulation models to better capture these longer-term outcomes and assess the sustainability of benefits as more data becomes available.

Next, the use of unlinked datasets limited our ability to model individual-level care trajectories across health care settings. This required us to make assumptions about the transitions between primary and specialist care, which may not fully reflect real-world complexity, such as mismatches or care discontinuities. While efforts were made to ground these assumptions in local clinical practice, future studies should aim to incorporate integrated, patient-level datasets to more accurately capture care pathways and evaluate outcomes.

Also, while implementation and migration costs were not included in this analysis, this reflects the practical context of Singapore’s health system, which has already committed to a nationwide transition to NGEMR. Thus, we focused on evaluating the incremental costs through bill sizes and benefits of NGEMR relative to EMR in the post-implementation phase. Future evaluations could adopt a broader cost perspective (eg, the total cost of ownership) to inform system-wide investment decisions in countries still considering such transitions.

Besides that, assumptions in parameterizing the model can introduce uncertainty that may affect the generalizability of the simulation results. While we attempted to mitigate this through validation with clinical stakeholders and sensitivity analyses, we acknowledge that more complex modeling approaches could enhance reliability, such as using linked patient-level datasets, where available. Future research should explore microsimulation modeling to capture individual heterogeneity more accurately. The model may also overestimate the cost savings from reduced duplicate testing, assuming that most repeated tests shortly after referral are redundant. In clinical practice, repeat testing may be necessary to monitor disease progression, especially if there is a delay between the initial test and the specialist consultation. The baseline estimate for reducing duplicate testing was derived from international literature, as local postimplementation data were unavailable. While care was taken to choose studies from health care systems with similar characteristics, this may limit the generalizability of the results to Singapore. Future analyses could benefit from more granular data on timing and clinical indications and incorporate real-world local data as they become available through ongoing NGEMR rollout evaluations.

Furthermore, this study does not explicitly incorporate differences in long-term maintenance or cybersecurity costs between EMR and NGEMR. Given that NGEMR systems are more integrated and support broader data sharing across institutions, they may be associated with increased risks of data breaches or higher costs for maintaining secure, compliant infrastructure. While such costs are often borne centrally or through capital budgets, their potential impact on cost-effectiveness and system sustainability should be addressed in future evaluations.

### Conclusion

NGEMR is cost-effective in the Singapore context when compared with EMR, provided that NGEMR can effectively reduce the number of duplicate tests and reduce waiting time, thus lowering the probability of admission after visiting a specialist. To evaluate the long-term cost-effectiveness of NGEMR, the waiting time to see a specialist and the probability of having duplicate tests are the key parameters to be monitored. Future research focusing on various benefits of NGEMR, such as reducing excessive tests and adverse drug events, is recommended to comprehensively understand the impact of NGEMR implementation in Singapore.

## Appendix

Multimedia Appendix 1 Comparison between EMR and NGEMR. EMR: Electronic Medical Record; NGEMR: Next Generation Electronic Medical Record.

Multimedia Appendix 2 CHEERS 2022 checklist. CHEERS: Consolidated Health Economic Evaluation Reporting Standards.

Multimedia Appendix 3 Interrupted-time series analysis of average waiting time (in days) from primary care referral in National University Polyclinics to specialist outpatient clinics after the implementation of NGEMR. NGEMR was implemented in the primary care setting at National University Polyclinics on 16 November 2020. NGEMR: Next Generation Electronic Medical Record.

Multimedia Appendix 4 Characteristics of patients vising primary care facility (NUP) and specialist care facility (SOCs) stratified by pre- and post-NGEMR periods. NUP: National University Polyclinics; SOC: Specialist outpatient clinics NGEMR: Next Generation Electronic Medical Record.

## Abbreviations

CHEERS: Consolidated Health Economic Evaluation Reporting Standards
ED: emergency department
EMR: electronic medical record
GDP: gross domestic product
ICER: incremental cost-effectiveness ratio
NGEMR: Next Generation Electronic Medical Record
NTFGH: Ng Teng Fong General Hospital
NUP: National University Polyclinics
PSA: probabilistic sensitivity analysis
QALY: quality-adjusted life year
S$: Singapore dollars
SOC: specialist outpatient clinic

## Data Availability

The datasets analyzed during this study are not publicly available due to confidentiality reasons, but are available from the corresponding author on reasonable request.
