# Supplementary material for: Economic Evaluation of the Next Generation Electronic Medical Records in Singapore: Cost-Utility Analysis
Source: J Med Internet Res. 2025 Jun 11;27:e70484. doi: 10.2196/70484 (PMC12198694; doi:10.2196/70484)
Supplement: Multimedia Appendix 1 [file jmir_v27i1e70484_app1.docx]

**Supplementary Appendix 1**. Comparison between EMR and NGEMR

|  | **EMR** | **NGEMR** |
| --- | --- | --- |
| **Operational** |  |  |
| Patient demographics | Primary and Specialist care use separate patient demographic database. Patient contact information may contain outdated data in some institutions. | Primary and Specialist care use the same patient demographic database, reducing need to re-register patient if patient has already been registered in the database. Patient contact details are shared and all institutions can contribute to accuracy of data. |
| Referral clarifications and scheduling | Specialist care has to use other means (e.g. telephone calls) to communicate with primary care referral operations team to clarify about referrals. | Specialist care is able to use NGEMR to send messages to primary care referral operations team to clarify about referrals.  Specialist team is able to see Primary care referral orders and schedule them in NGEMR. |
| **Clinical** |  |  |
| Viewing of Specialist EMR records | Primary care doctors can view Specialist records through another system, which requires launching another software with multiple clicks. | Primary care doctors can view Specialist records within the same system. |
| Ability to edit a shared patient record that benefits patient care in all institutions | Primary care doctors is unable to edit shared patient records. | Primary care can now edit shared records such as Problem List, long-term medications, which continue to be visible to other NGEMR institutions. |
| **Analytics** |  |  |
| Analysing data warehouses of each institution | Each institution stores patients’ data in their own warehouse in different formats. To analyse data collectively, they need to be merged. | Patients’ data is stored in a common warehouse in the same format, making it easier to analyse collective data. |
| **Conflicts** |  |  |
| EMR features intended for an institution can result in unwanted new features being exposed to other institutions. | Primary and Specialist care have customised systems, with features tailored to their workflow requirements. Fewer resources need to be employed to ensure that systems don't conflict with each other. Maintenance cost of each system is lower, but each system requires a small maintenance team contributing to multiple small teams maintaining these systems. | New features available in NGEMR, as well as subsequent newly-created enhancements, meant for some institutions, might be imposed on other institutions without these requirements. To avoid this, more resources have to be employed to avoid such conflicting implementations. Maintenance cost of an integrated system is higher - each institution requires a larger team contributing to a single large team maintaining NGEMR. |
