# Supplementary material for: Economic Evaluation of the Next Generation Electronic Medical Records in Singapore: Cost-Utility Analysis
Source: J Med Internet Res. 2025 Jun 11;27:e70484. doi: 10.2196/70484 (PMC12198694; doi:10.2196/70484)
Supplement: Multimedia Appendix 2 [file jmir_v27i1e70484_app2.docx]

**Supplementary Appendix 2.** CHEERS 2022 Checklist

|  | **Item** | **Guidance for reporting** | **Reported**  **in section** |
| --- | --- | --- | --- |
| **TITLE** | | | |
| Title | 1 | Identify the study as an economic evaluation and specify the interventions being compared. | Title |
| **ABSTRACT** | | | |
| Abstract | 2 | Provide a structured summary that highlights context, key methods, results and alternative analyses. | Abstract |
| **INTRODUCTION** | | | |
| Background and objectives | 3 | Give the context for the study, the study question and its practical relevance for decision making in policy or practice. | Introduction |
| **METHODS** | | | |
| Health economic analysis plan | 4 | Indicate whether a health economic analysis plan was developed and where available. | Methods |
| Study population | 5 | Describe characteristics of the study population (such as age range, demographics, socioeconomic, or clinical characteristics). | Supplementary Table 1 |
| Setting and location | 6 | Provide relevant contextual information that may influence findings. | Methods |
| Comparators | 7 | Describe the interventions or strategies being compared and why chosen. | Methods |
| Perspective | 8 | State the perspective(s) adopted by the study and why chosen. | Methods |
| Time horizon | 9 | State the time horizon for the study and why appropriate. | Methods |
| Discount rate | 10 | Report the discount rate(s) and reason chosen. | Not applicable as we evaluated NGEMR after one year of implementation |
| Selection of outcomes | 11 | Describe what outcomes were used as the measure(s) of benefit(s)  and harm(s). | Methods |
| Measurement of outcomes | 12 | Describe how outcomes used to capture benefit(s) and harm(s). | Methods, input parameters |
| Valuation of outcomes | 13 | Describe the population and methods used to measure and value outcomes. | Methods, input parameters |
| Measurement and valuation of resources and costs | 14 | Describe how costs were valued. | Methods, input parameters |
| Currency, price date, and conversion | 15 | Report the dates of the estimated resource quantities and unit costs, plus the currency and year of conversion. | Methods, input parameters |
| Rationale and description of model | 16 | If modelling is used, describe in detail and why used. Report if the model is publicly available and where it can be accessed. | Methods, modelling structure |
| Analytics and assumptions | 17 | Describe any methods for analysing or statistically transforming data, any extrapolation methods, and approaches for validating any model used. | Methods, cost-effectiveness analysis and uncertainty analysis |
| Characterizing heterogeneity | 18 | Describe any methods used for estimating how the results of the study. | Not applicable |
| Characterizing distributional effects | 19 | Describe how impacts are distributed across different individuals or adjustments made to reflect priority populations. | Not applicable |
| Characterizing uncertainty | 20 | Describe methods to characterize any sources of uncertainty in the analysis. | Methods, cost-effectiveness analysis and uncertainty analysis |
| Approach to engagement with patients and others affected by the study | 21 | Describe any approaches to engage patients or service recipients, the general public, communities, or stakeholders (e.g., clinicians or payers) in the design of the study. | Not applicable |
| **RESULTS** | | | |
| Study parameters | 22 | Report all analytic inputs (e.g., values, ranges, references) including uncertainty or distributional assumptions. | Table 1 |
| Summary of main results | 23 | Report the mean values for the main categories of costs and outcomes of interest and summarise them in the most appropriate overall measure. | Table 2 |
| Effect of uncertainty | 24 | Describe how uncertainty about analytic judgments, inputs, or projections affect findings. Report the effect of choice of discount rate and time horizon, if applicable. | Figures 2A-B, 3, and 4 |
| Effect of engagement with patients and others affected by the study | 25 | Report on any difference patient/service recipient, general public, community,  or stakeholder involvement made to the approach or findings of the study. | Not applicable |
| **DISCUSSION** | | | |
| Study findings, limitations, generalizability, and current knowledge | 26 | Report key findings, limitations, ethical or equity considerations not captured, and how these could impact patients, policy, or practice. | Discussion |
| **OTHER RELEVANT INFORMATION** | | | |
| Source of funding | 27 | Describe how the study was funded and any role of the funder in the identification, design, conduct, and reporting of the analysis. | Source of funding |
| Conflict of interest | 28 | Report authors conflicts of interest according to journal or International Committee of Medical Journal Editors requirements. | Conflict of interest |
