# Supplementary figures and images for: Economic Evaluation of the Next Generation Electronic Medical Records in Singapore: Cost-Utility Analysis
Source: J Med Internet Res. 2025 Jun 11;27:e70484. doi: 10.2196/70484 (PMC12198694; doi:10.2196/70484)

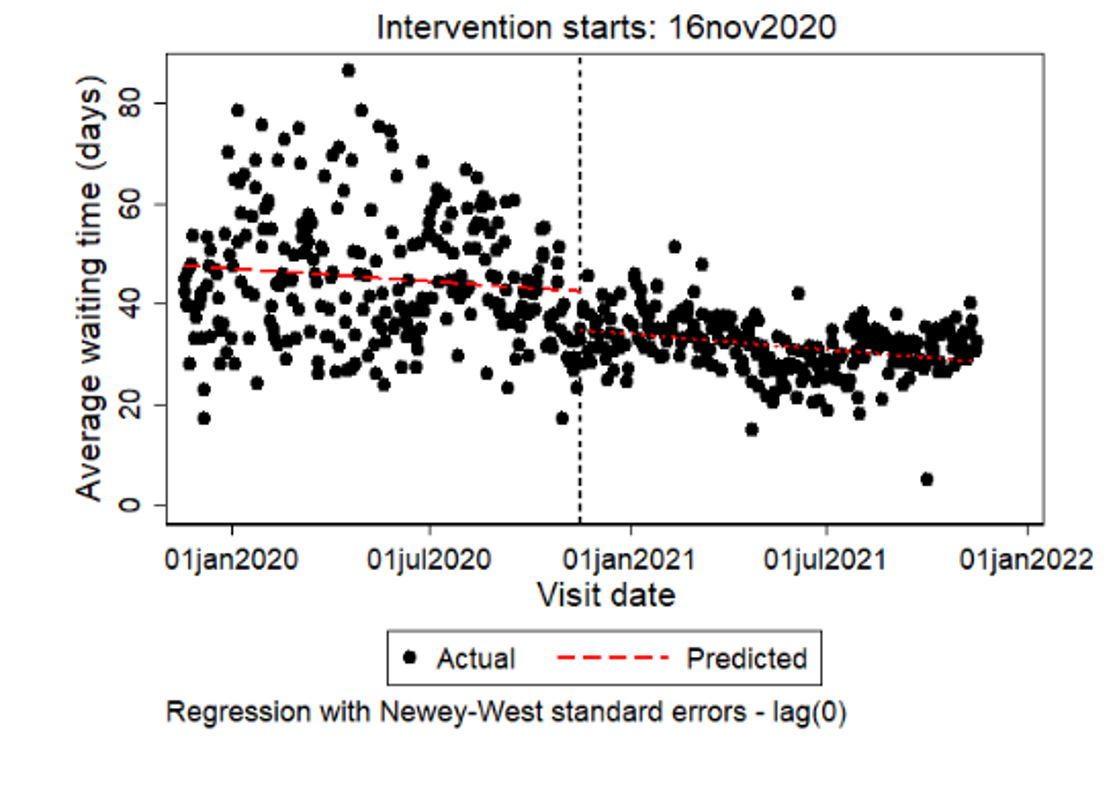

Supplement: Multimedia Appendix 3 [file jmir_v27i1e70484_app3.png]
