# Supplementary material for: Economic Evaluation of the Next Generation Electronic Medical Records in Singapore: Cost-Utility Analysis
Source: J Med Internet Res. 2025 Jun 11;27:e70484. doi: 10.2196/70484 (PMC12198694; doi:10.2196/70484)
Supplement: Multimedia Appendix 4 [file jmir_v27i1e70484_app4.docx]

**Supplementary Appendix 4**. Characteristics of patients vising primary care facility (NUP) and specialist care facility (SOCs) stratified by pre- and post-NGEMR periods^a^

|  | **Pre-NGEMR** | **Post-NGEMR** | **P-value** |
| --- | --- | --- | --- |
| **Primary care, before matching** |  |  |  |
| Number of patients^b^ | 229605 | 154918 | **-** |
| Polyclinic, n (%) |  |  | < 0.001 |
| Bukit Batok | 47275 (20.6) | 29560 (19.1) |  |
| Choa Chu Kang | 56066 (24.4) | 35208 (22.7) |  |
| Clementi | 38540 (16.8) | 27362 (17.7) |  |
| Jurong | 51024 (22.2) | 35933 (23.2) |  |
| Pioneer | 36700 (16.0) | 26855 (17.3) |  |
| Type of visit, n (%) |  |  | < 0.001 |
| Acute | 72310 (31.5) | 58265 (37.6) |  |
| Chronic | 157295 (68.5) | 96653 (62.4) |  |
| Charge per visit (S$), median (IQR) | 65.4 (50.5, 122.5) | 68.3 (53.1, 129.8) | < 0.001 |
| **Primary care, after matching^c^** |  |  |  |
| Number of patients^b^ | 155116 | 154918 | **-** |
| Polyclinic, n (%) |  |  | < 0.001 |
| Bukit Batok | 31633 (20.4) | 29560 (19.1) |  |
| Choa Chu Kang | 37253 (24.0) | 35208 (22.7) |  |
| Clementi | 25942 (16.7) | 27362 (17.7) |  |
| Jurong | 34491 (22.2) | 35933 (23.2) |  |
| Pioneer | 25797 (16.6) | 26855 (17.3) |  |
| Type of visit, n (%) |  |  | 0.855 |
| Acute | 58289 (37.6) | 58265 (37.6) |  |
| Chronic | 96827 (62.4) | 96653 (62.4) |  |
| Charge per visit (S$), median (IQR) | 63.5 (50.3, 117.5) | 68.3 (53.1, 129.8) | < 0.001 |
| **SOCs, service level patient data** |  |  |  |
| Number of patients^b^ | 13650 | 17920 | **-** |
| Age (years), median (IQR) | 59 (45, 69) | 58 (44, 68) | 0.002 |
| Male, n (%) | 7682 (56.3) | 9521 (53.1) | < 0.001 |
| Ethnicity, n (%) |  |  | < 0.001 |
| Chinese | 10135 (74.2) | 12852 (71.7) |  |
| Malay | 1659 (12.2) | 2267 (12.7) |  |
| Indian | 1217 (8.9) | 1781 (9.9) |  |
| Others | 639 (4.7) | 1020 (5.7) |  |
| Referral source, n (%) |  |  | < 0.001 |
| Bukit Batok | 199 (1.5) | 2186 (12.2) |  |
| Choa Chu Kang | 8 (0.1) | 1356 (7.6) |  |
| Clementi | 55 (0.4) | 1235 (6.9) |  |
| Jurong | 13117 (96.1) | 9610 (53.6) |  |
| Pioneer | 271 (2.0) | 3533 (19.7) |  |
| Waiting time (days), median (IQR)^d^ | 28 (12, 55) | 23 (7, 51) | < 0.001 |
| **SOCs, financial data** |  |  |  |
| Number of patients^b^ | 21827 | 16828 | **-** |
| Bill per visit (S$), median (IQR)^e^ | 89.9 (74.0, 141.0) | 94.7 (74.0, 141.0) | < 0.001 |
| Charge per visit (S$), median (IQR)^f^ | 134.9 (111.0, 211.5) | 142.1 (111.0, 211.5) | < 0.001 |

^a^As NGEMR was implemented across the health system and in phases, specialist care was from 28 February 2020, whereas primary care was implemented on 16 November 2020. Thus, patients visiting specialist care from 28 February 2019 to 27 February 2021 were included. However, as the NUP dataset before 1 September 2019 was unavailable, we included only patients visiting NUP from 1 September 2019 to 31 January 2020 to ensure an equal period before and after the implementation of NGEMR.

^b^The number of patients was identified using unique patient identification and visit dates.

^c^Patients visiting NUP in different periods were matched using the type of visit, either acute or chronic.

^d^The waiting time was defined as the number of days between the primary care visit date and the specialist visit date.

^e^The bill per visit indicated the post-subsidy cost per visit (1).

^f^Due to data unavailability, the charge per visit (pre-subsidy cost) was calculated by inflating the bill per visit by 50%. We assumed the 50% subsidy rate (2) based on the 2020 median household monthly income per person of S$2,463 (3)

| **References**  1. Ministry of Health. Bill Presentment for Public Healthcare Institutions (PHIs) 2021 [updated 19 Apr 2021. Available from: <https://www.moh.gov.sg/cost-financing/bill-presentment-for-public-healthcare-institutions>.  2. Ministry of Health. Subsidies for acute inpatient care at public healthcare institutions 2023 [updated 05 Oct 2023. Available from: <https://www.moh.gov.sg/healthcare-schemes-subsidies/subsidies-for-acute-inpatient-care-at-public-healthcare-institutions>.  3. Department of Statistics. Census of population 2020 statistical release 2: households, geographic distribution, transport and difficulty in basic activities. Singapore; 2020. |  |
| --- | --- |
